# Implications of NKG2A in immunity and immune-mediated diseases

**DOI:** 10.3389/fimmu.2022.960852

**Published:** 2022-08-10

**Authors:** Xiaotong Wang, Huabao Xiong, Zhaochen Ning

**Affiliations:** ^1^ Institute of Immunology and Molecular Medicine, Jining Medical University, Jining, China; ^2^ Jining Key Laboratory of Immunology, Jining Medical University, Jining, China

**Keywords:** NKG2A, cancer immunotherapy, viral infections, autoimmune diseases, HLA-E

## Abstract

In recent studies, NKG2A is revealed to be a key immune checkpoint for both natural killer (NK) cells and CD8^+^ T cells. It form heterodimer receptors with CD94, and targets the peptide-presenting human leukocyte antigen-E (HLA-E) molecules. Upon crosslinking, NKG2A/CD94 delivers inhibitory signals for NK cells and CD8^+^ T cells, while blocking NKG2A can effectively unleash functions of these cytotoxic lymphocytes. The interaction between NKG2A and HLA-E contributes to tumor immune escape, and NKG2A-mediated mechanisms are currently being exploited to develop potential antitumor therapeutic strategies. In addition, growing evidence shows that NKG2A also plays important roles in other immune-related diseases including viral infections, autoimmune diseases, inflammatory diseases, parasite infections and transplant rejection. Therefore, the current work focuses on describing the effect of NKG2A on immune regulation and exploring its potential role in immune-mediated disorders.

## Introduction

NKG2A is a member of C-type lectin superfamily (1, 2). Its gene is localized in the natural killer (NK) complex on chromosome 12 and consists of seven exons (3). NKG2A is a single-pass type II integral membrane glycoprotein that contains the cytoplasmic, transmembrane as well as extracellular lectin-like domains (4). The intracellular portion has two ITIMs, which are involved in inhibitory signal transduction (5). NKG2A expression can be detected in cytotoxic lymphocytes, including most NK cells and a subset of CD8^+^ T cells (6). It is found to be expressed as a heterodimer with CD94, which also belongs to the C-type lectin superfamily (7). The ligands of NKG2A/CD94 heterodimeric receptor are non-classical MHC class I molecules, human leukocyte antigen (HLA)-E in humans and Qa-1 in mice (8). HLA-E is lowly expressed on almost all cell surfaces and displays limited polymorphism (9). Peptides that presented by HLA-E are derived from the leader sequences of the classical MHC class I molecules such as HLA­A, HLA­B and HLA­C (10). Engagement of NKG2A/CD94 receptor with peptide-presenting HLA-E results in the phosphorylation of ITIMs in NKG2A. Phosphorylated ITIMs are responsible for recruiting and activating intracellular phosphatase SHP-1 as well as SHP-2, thus suppressing the activation signals generated by activating receptors such as T cell receptor (TCR) and NKG2D (11). In contrast to classical HLA molecules, which are commonly lost (12), the expression of HLA-E is generally elevated within tumor cells (13). Similar to other immune checkpoint molecules, NKG2A is exploited by tumor cells to achieve immune evasion. In addition, disrupting the interaction of NKG2A with its ligands is shown to be effective in enhancing antitumor immune responses (6, 14, 15). The overexpression of HLA-E is also observed in viral-infected cells, and the NKG2A-HLA-E axis is proved to exert a vital role in viral infection (16). Notably, NKG2A expression is found to be correlated with disease severity in coronavirus disease 2019 (COVID-19) patients (17–20). Apart from that, NKG2A is also involved in the pathological process of other immune-mediated disorders, such as autoimmune diseases, inflammatory diseases, parasite infections and transplant rejection. These findings indicate that NKG2A is a new therapeutic target for managing a variety of immune-mediated disorders. Herein, we review the existing knowledge about NKG2A mediated immune regulation and discuss the implications of NKG2A-targeted immunotherapeutic strategies.

## Effects of NKG2A in immunocytes

### NKG2A and NK cells

Approximately half of the human peripheral blood NK cells display NKG2A expression (21–23). Its expression is mostly observed in CD56^bright^ immature NK cells and is decreased with stepwise maturation of the NK cells (24). There exits a negative correlation between the expression of NKG2A and killer cell immunoglobulin­like receptors (KIRs), which is implicated in the differentiation process of NK cells (25, 26). Multiple cytokines including interleukin (IL)-21, IL-15, IL-12, IL-10 and transforming growth factor-β (TGF-β) are able to induce the expression of NKG2A in NK cells (27–30). Upon ligands binding, NKG2A/CD94 receptors deliver signals that suppress NK cell functions, while disrupting the interaction of NKG2A/CD94 with Qa-1 or HLA-E activates the cytotoxic activity of NK cells (31–33).

The inhibitory NK receptors for HLA act a pivotal part in the education of NK cells, thereby greatly affecting mature NK cell responsiveness (34). At least one inhibitory NK receptors specific for “self” HLA-I haplotype need to be expressed on mature NK cells for recognizing target cells as well as preventing the activation of NK cells against autologous cells (35). The lack of inhibitory NK receptors for HLA renders NK cells hyporesponsive (36), while educated NK cells that expressing these receptors show higher responsiveness (37). Several studies have shown that NKG2A is required for the education of NK cells (38–40). NKG2A-educated human NK cells were more effective at killing target cells and showed a more dynamic migration behavior (41). NKG2A-educated mouse uterine NK cells were found to be more functionally competent in response to NK1.1 crosslinking (42). Highton et al. reported that NKG2A-educated human NK cells displayed improved responsiveness and metabolic resilience compared to KIRs-educated counterparts (43).

NK cells must maintain an appropriate level of NKG2A expression on the cell surface so as not to destroy normal autologous cells (44). NKG2A can be reused through a relatively rapid recycling process, enabling continuous availability of NKG2A on the cell surface. This recycling process requires energy and the cytoskeleton, but does not require functional ITIMs (44). The interaction of NKG2A/CD94 receptor with its ligands does not affect this recycling process and the expression of NKG2A/CD94 within NK cells (25). According to the fluorescent recovery after photobleaching (FRAP) analysis, most NKG2A/CD94 molecules on the plasma membrane exist in a free-moving form. NKG2A/CD94 is enriched at the contact site after crosslinking, and this enrichment is achieved through lateral diffusion in plasma membrane rather than synthesis of new proteins (45).

### NKG2A and T cells

NKG2A is also expressed in T cells, especially CD8^+^ T cells. NKG2A expression in CD8^+^ T cells is found to be highly regulated, differing from its expression pattern in NK cells. NKG2A is barely expressed in CD8^+^ T cells of healthy individuals, but upregulated in tumor lesions and during chronic viral infection (46, 47). The expression of NKG2A in CD8^+^ T cells can be modulated a number of cytokines such as IL-23, IL-21, IL-15, IL-10, IL-6, IL-4, IL-2 and TGF-β (48–51). In addition, NKG2A expression can be induced by TCR engagement, and is acquired after antigen encounter. Cytotoxic T lymphocyte (CTL) clones sharing the same antigen specificity have the same NKG2A expression pattern, indicating that TCR antigenic specificity dictates the expression of NKG2A (52). NKG2A marks a special CD8^+^ T cells subset harboring tissue-resident and terminally exhausted features (6, 53–55). Similar to its function in NK cells, NKG2A/CD94 receptor engagement delivers inhibitory signals to CD8^+^ T cells, thus inhibiting the cytotoxic activity (55). Besides, NKG2A has also been found to be expressed in human CD4^+^ T cells. The expression of NKG2A/CD94 was observed in anti-CD3 monoclonal antibody (mAb) activated CD4^+^ T cells under TGF-β and IL-10 treatment. Moreover, NKG2A/CD94 was functional in CD4^+^ T cells and could inhibit TCR mediated tumor necrosis factor-α (TNF-α) and interferon-γ (IFN-γ) secretion (56).

## The functions of NKG2A in immunopathological settings

### NKG2A and tumors

Overexpression of HLA-E is observed in various types of tumors, including solid tumors as well as hematological malignancies (57–63). Meanwhile, HLA-E overexpression predicts a poor prognostic outcome in patients with ovarian, liver, gynecologic, glioblastoma, colorectal, breast, gastric, kidney, esophageal, pancreatic, lung, and head and neck cancer (64–72). HLA-E overexpression may be caused by the interaction of tumor cells with tumor microenvironment (TME), and there was evidence that IFN-γ produced by tumor-reactive immunocytes contributed to the upregulation of HLA-E within tumor cells (73, 74). However, the underlying mechanism is not completely clear so far. Since overexpressed HLA-E functions to inhibit the cytotoxicity of cytotoxic lymphocytes, the blockade of NKG2A-HLA-E axis may possibly enhance the cell-based immunotherapeutic efficacy. In addition, according to multiple studies, NKG2A is also overexpressed in tumor-infiltrating cytotoxic lymphocytes in many types of tumors (55, 66, 75). The increase in the number of NKG2A^+^ tumor infiltrating lymphocytes (TILs) is correlated with a poor prognosis in patients undergoing colorectal, ovary and liver cancer (65, 72). Moreover, there is a growing body of evidence that NKG2A-HLA-E axis contributes to tumor immune escape. Sheu BC et al. found human cervical cancer cells could upregulate NKG2A expression in CD8^+^ T cells through an IL-15-dependent mechanism, thus abrogating the antitumor cytotoxicity of TILs (76). The cytotoxic effects of human NK cells and CD8^+^ T cells on HLA-E expressing B-lymphoblastoid cells were enhanced through RNAi-mediated inhibition of NKG2A expression (77). Kamiya and colleagues knocked out NKG2A protein expression in human peripheral blood NK cells achieved by retroviral transduction of NKG2A blocker, thus generating NKG2A^null^ NK cells. They further found NKG2A^null^ NK cells showed higher cytotoxic activity against HLA-E expressing tumor cells in immunodeficient mice (31). According to the results of the *in vitro* experiment, blocking NKG2A in human NK cells by the humanized anti-NKG2A antibody monalizumab was sufficient for improving the dysfunction of NK cells in chronic lymphocytic leukemia (CLL) (78). Salomé et al. showed that NKG2A was highly expressed in type 1 innate lymphoid cells (ILC1s) of acute myeloid leukemia (AML) patients. Moreover, the cytotoxicity of NKG2A^+^ ILC1s was impaired when encountering HLA-E-expressing leukemic targets (79). Collectively, the above results indicate that it is worthwhile to develop NKG2A blockade strategies for immunotherapy in cancer patients. However, clinical and preclinical studies showed that blocking NKG2A alone did not appear to be effective for tumor therapy. Monalizumab monotherapy showed very little clinical activity in patients with gynecologic cancers (80). Consistently, data from pre-clinical research suggested that anti-NKG2A mAb alone showed no effect on subcutaneous tumor xenografts in mice (14, 55). Regardless of the above drawbacks, monalizumab is still useful in combination with other immunotherapies. As demonstrated by preliminary data in microsatellite stable colorectal cancer (CRC) patients who typically do not respond to programmed death-ligand 1 (PD-L1)/programmed cell death-1 (PD-1)-based therapy, the combination of monalizumab and durvalumab (an anti-PD-L1 mAb) showed clinical efficacy and safety (81). Andre´ et al. also showed that combined blockade of PD-L1/PD-1 and NKG2A enhanced anticancer immunity in mouse lymphoma tumor models (14). According to the phase II trials interim results, the objective response rate (ORR) in head and neck squamous cell carcinoma (HNSCC) patients receiving monalizumab and cetuximab (an anti-EGFR blocking mAb) combination therapy was 31%, which was superior to previous data obtained from cetuximab monotherapy. The action of the above combinational therapy is probably mediated *via* NKG2A^+^ NK cells, rather than NKG2A^+^ CD8^+^ T cells (14). Additionally, according to van Hall’s group study based on four mouse models of solid tumors, the antitumor activity of CD8^+^ T cells responding to peptide vaccination was restored through blocking NKG2A-Qa-1 axis using blocking antibodies or genetic knockout (55). In addition, there are a number of ongoing clinical trials with monalizumab for the treatment of tumors, as shown in **Table 1**.

**Table 1:** Ongoing clinical trials with monalizumab for the treatment of tumors.

Clinical trial	Phase	Drug	Disease	Participants	Status	First Posted
NCT05414032	II	Monalizumab Cetuximab	Locoregionally advanced HNSCC	200	Not yet recruiting	June 10, 2022
NCT05221840	III	Monalizumab Durvalumab Oleclumab Placebo	Non-small cell lung cancer (NSCLC)	999	Recruiting	February 3, 2022
NCT05061550	II	Monalizumab Durvalumab Oleclumab	NSCLC	140	Recruiting	September 29, 2021
NCT04590963	III	Monalizumab Cetuximab	HNSCC	624	Recruiting	October 19, 2020
NCT04307329	II	Monalizumab Trastuzumab	Breast cancer	38	Recruiting	March 13, 2020
NCT03833440	II	Monalizumab Durvalumab Oleclumab Ceralasertib Docetaxel	NSCLC	120	Recruiting	February 7, 2019
NCT03822351	II	Monalizumab Durvalumab Oleclumab	Stage III NSCLC	188	Active, not recruiting	January 30, 2019
NCT03088059	II	Monalizumab Afatinib Palbociclib Durvalumab Niraparib INCAGN01876 standard of care	Recurrent or metastatic HNSCC	340	Recruiting	March 23, 2017
NCT02921685	I	Monalizumab	Hematologic malignancies	18	Unknown	October 3, 2016
NCT02643550	I/II	Monalizumab Cetuximab Anti-PD(L)1	Recurrent or metastatic HNSCC	143	Active, not recruiting	December 31, 2015

### NKG2A and viral infections

Altering MHC molecules expression on the infected cell surface is one of the mechanisms that mediate viral immune escape. HLA-E and Qa-1 usually show overexpression on the virus infected cell surfaces and are able to bind peptides derived from viral proteins. HLA-E overexpression was observed in hepatic antigen-presenting cells (APCs) of hepatitis C virus (HCV) infected patients. HLA-E could bind to the viral peptide HCV core aa35–44 and present it on the cell surface, where it interacted with NKG2A/CD94 heterodimers, thereby resulting in immunosuppression (82). HCV infection induced Qa-1 expression in mouse hepatocytes. Abrogation of either NKG2A or Qa-1 signaling was shown to enhance NK function and promote NK cell-dependent HCV clearance (33). Similar findings were obtained during human cytomegalovirus (HCMV) infection. Glycoprotein UL40 encoded by HCMV could bind to HLA-E and interact with NKG2A/CD94 receptors, thereby inhibiting human NK cell activity and leading to immune evasion (83–85). HLA-E expression was enhanced in lymphocytes of human immunodeficiency virus (HIV) infected patients, and viral peptide HIV p24 aa14-22-loaded HLA-E could inhibit NK cell cytotoxic activity by binding to NKG2A (86). However, according to van Stigt Thans et al., HIV-1 downregulated the expression of HLA-E on the surface of infected primary human CD4^+^ T cells (87). During human papillomavirus (HPV) infection, the decreased expression of classical HLA class I molecules and overexpression of HLA-E were observed. In addition, HLA-E overexpression was associated with the decreased cytotoxicity of NK cells, which was most likely achieved through the interaction with NKG2A/CD94 receptors (88). Interestingly, a recent study showed that not all peptides presented by HLA-E could bind to NKG2A and thus exert inhibitory effects. As discovered by Mbiribindi B and colleagues, human NKG2A^+^ NK cells was able to recognize and respond to Epstein-Barr virus (EBV) infected autologous B cells. Further studies showed that EBV latent cycle protein-derived peptides impaired the recognition of NKG2A, despite being presented by HLA-E, thereby leading to the absence of inhibition (89).

The expression of NKG2A in NK cells is also generally increased during viral infection. The dysfunction of NK cells and T cells was observed in chronic hepatitis B (CHB) patients, along with the overexpression of inhibitory receptors including PD-1 and NKG2A (90). Hepatitis B e-antigen (HBeAg) was able to induce IL-10 secretion within regulatory T cells (Tregs), thus upregulating NKG2A expression in NK cells of CHB patients (91). The overexpressed NKG2A severely impaired the cytotoxicity of NK cells during HBV infection, which could be restored through the blockade of NKG2A-HLA-E axis (90, 91). Among the EBV reactivation and EBV-chronic graft-versus-host disease (GvHD) patients after hematopoietic stem cell transplantation (HSCT), the frequency of NKG2A^+^CD56^dim^ NK cells was significantly increased in peripheral blood (92). According to Hendricks et al., the coinfection of cytomegalovirus (CMV) and EBV led to NKG2A^+^CD56^dim^ NK cell expansion (93). Interestingly, there was a study reveal an innovative viral immune evasion mechanism. According to Wang et al., rodent herpesvirus Peru could counteract mouse NK cell activation by encoding a Qa-1 like protein *via* RNA splicing (94).

In addition to NK cells, NKG2A also has an impact on CD8^+^ T cell-mediated antiviral immunity. During polyoma virus infection in mice, NKG2A expression was enhanced in antiviral CD8^+^ T cells, thereby leading to the decrease of antigen-specific cytotoxicity in the process of virus-mediated oncogenesis and viral clearance (95). During ectromelia virus infection, NKG2A functioned intrinsically within mouse virus-specific CD8^+^ T cells for limiting excessive activation (96). However, NKG2A does not appear to affect CD8^+^ T cell-mediated antiviral immunity in all types of viral infections. Miller et al. found NKG2A/CD94 heterodimers showed no inhibitory effect on CD8^+^ T cell activity during lymphocytic choriomeningitis virus (LCMV) infection in mice (97).

Significantly, recent studies have highlighted a key role of NKG2A in the infection with severe acute respiratory syndrome coronavirus 2 (SARS-CoV-2). The expression of NKG2A was enhanced in peripheral NK cells and CD8^+^ T cells of COVID-19 patients and was correlated with disease severity (17–20). Interestingly, the NKG2A^+^ cytotoxic lymphocytes proportion was reduced among recovered patients (17). Further research has shown that the NKG2A expression is found to be regulated by SARS-CoV-2 spike 1 protein (SP1). The results of *in vitro* experiments showed that coculture with SP1-transfected lung epithelial cells led to NKG2A overexpression and reduced degranulation in NK cells (98). In addition, NKG2A was also overexpressed in NK cells isolated from bronchoalveolar lavage fluid (BALF) of COVID-19 patients with acute respiratory distress syndrome (ARDS), and the expression level was even higher than that in blood cells (19). In general, the NKG2A-HLA-E axis can be exploited by viruses to limit the activity of cytotoxic lymphocytes, thereby contributing to viral immune escape (**Table 2**).

**Table 2:** Roles of NKG2A-HLA-E axis in viral infections.

Condition	Roles of NKG2A-HLA-E axis
HCV	HCV Core aa35-44 could bind to HLA-E and stabilize its membrane expression, thus inhibiting NK cell cytotoxicity by the interaction with NKG2A (82). HCV infection induced Qa-1 expression in mouse hepatocytes. Blocking NKG2A-Qa-1 axis was able to restore the function of NK cells and promote virus clearance (33).
HCMV	HCMV-encoded glycoprotein UL40 could bind to HLA-E and interact with NKG2A/CD94 receptors, thereby inhibiting NK cell activation (83–85).
HIV	HIV p24 aa14-22-loaded HLA-E impaired NK cell function by binding to NKG2A (86).
HPV	HLA-E overexpression was observed in cervical biopsies of women infected with HPV and was associated with the inhibition of NK cell cytotoxicity (88).
EBV	EBV latent cycle protein-derived peptides could bind to HLA-E, but impair the recognition of NKG2A expressed by NK cells, thereby leading to the absence of inhibition (89). In EBV reactivation and EBV-chronic GvHD patients after HSCT, the frequency of NKG2A^+^CD56^dim^ NK cell population was increased in peripheral blood (92).
HBV	NKG2A overexpression was observed in NK cells of CHB patients and severely impaired the cytotoxicity of NK cells during HBV infection (90, 91).
Polyoma virus	NKG2A overexpression impaired the cytotoxicity of antiviral CD8^+^ T cells in mice (95).
Ectromelia virus	NKG2A functioned intrinsically within mouse virus-specific CD8^+^ T cells to limit excessive activation (96).
LCMV	NKG2A/CD94 heterodimers showed no inhibitory effect on CD8^+^ T cell activity in mice (97).
SARS-CoV-2	NKG2A expression was increased in peripheral cytotoxic lymphocytes of COVID-19 patients and was correlated with the severity of disease (17–20).

### NKG2A and autoimmune diseases

NK cells are able to eliminate autoreactive T cells, while NKG2A expressed within NK cells functions to prevent this process (99–101). Hence the blockade of NKG2A-ligand interaction is an efficient approach to treat autoimmune diseases. NKG2A/CD94 receptor has been found to exert a critical influence on experimental autoimmune encephalomyelitis (EAE) by modulating T cell activity. Typically, the interaction of NKG2A with its ligands was essential for immunologic memory development and clonal expansion of autoreactive T cells, as well as contributed to the protection of activated CD4^+^ T cells from lysis by NKG2A^+^ NK cell. The blockade of NKG2A-Qa-1 axis could effectively promote the elimination of autoreactive T cells mediated by NK cells, thereby alleviating EAE in mice (102, 103). Consistently, in comparison with CD4^+^ T cells obtained from Qa-1 wild type mice, NK cells showed higher cytotoxic activity against activated CD4^+^ T cells isolated from Qa-1 deficient mice (102). As observed from the rheumatoid arthritis (RA) mouse model, blocking NKG2A accelerated NK cell mediated elimination of pathogenic T helper 17 (Th17) cells as well as follicular helper T (Tfh) cells, thus arresting disease progression (104).

The expression profile of NKG2A within NK cells varies among different autoimmune diseases. Compared with NK cells from healthy individuals, NK cells from systemic lupus erythematosus (SLE) patients showed lower cytotoxicity with enhanced NKG2A expression (105, 106). On the contrary, a decrease in NKG2A expression was observed in NK cells of Graves’ disease (57) and new-onset psoriasis (107) patients. There were reports that the expression of NKG2A in T cells was decreased in SLE patients (108) and rheumatoid arthritis (RA) patients who flared (109), indicating that lack of inhibitory signals might lead to T cell hyperactivation and the immunological disorders. However, this conclusion is not applicable for CD8^+^ Tregs. CD8^+^ Tregs function to suppress self-reactive CD4^+^ T cells activity, thereby alleviating EAE. Upon Binding to Qa-1, NKG2A/CD94 receptor functioned to inhibit CD8^+^ Tregs activity. Disrupting the interaction of NKG2A/CD94 with Qa-1 unleashed CD8^+^ Tregs activity and abolished EAE progression in mice (110). Besides, the overexpressed NKG2A in CD8^+^ Tregs of patients with relapsing multiple sclerosis (MS) may function to limit CD8^+^ Tregs activity and contribute to disease progression (111).

### NKG2A and other immune-related diseases

In addition to tumors, viral infections and autoimmune diseases, NKG2A is also involved in the pathological process of other immune-related diseases including inflammatory diseases, parasite infections and transplant rejection. NKG2A generally exerts immunosuppressive effects in inflammation. As reported by Hall and colleagues, NK cells inhibited the pro-inflammatory function of activated neutrophils through NKG2A-dependent mechanism in a DSS-induced colitis mouse model. Therefore, NKG2A played a protective role by inhibiting inflammation, while blocking NKG2A aggravated neutrophil-induced inflammation and tissue damage (112). In line with this, Zou and colleagues showed that the overexpressed NKG2A in NK cells exerted a vital function in suppressing neutrophil activation, thus alleviating DSS-induced colitis in mice (113). In celiac disease (CD) patients, CD-associated inflammation was marked by a decreased frequency of NKG2A^+^ natural killer T cells (NKT) and NKG2A^+^ NK cells, which might be involved in CD-associated tissue damage mediated by cytotoxic lymphocytes (114). Synovial NK cells from arthritis patients exhibited an activated phenotype and were capable of producing TNF-α and IFN-γ. Further, the secretion of these pro-inflammatory cytokines was increased under NKG2A blocking antibody treatment and decreased when NK cells encountered HLA-E expressed target cells, suggesting NKG2A can take part in the regulation of inflammatory response by controlling cytokines secretion in NK cells (115).

The immunosuppressive function of NKG2A has also been demonstrated in parasitic infection studies. In mice with alveolar echinococcosis (AE), which was caused by the infection of *Echinococcus multilocularis*, the overexpression of NKG2A was observed in NK cells and further led to reduced cytotoxicity by inhibiting IFN-γ secretion (116). Human cystic echinococcosis is caused by the larval stage of *Echinococcus granulosus sensu lato*. The expression of NKG2A and HLA-E was significantly increased within the hepatic cystic echinococcosis lesion, suggesting an inhibitory microenvironment (117). High expression of NKG2A was observed in human malaria-responsive NK cells (118) and γδ-T cells (119), which provided a homeostatic regulation mechanism for avoiding persistent activation. In addition, the expression of NKG2A was also found to be increased in human NK cells during *Toxoplasma gondii* (120) and *Gnathostoma spinigerum* infections (121). In contrast, during *Schistosoma japonicum* infection, NKG2A expression was significantly decreased in mouse NK cells (122) and NKT cells (123), which might serve as an activation mechanism.

GvHD is one of the major complications and causes of death in HSCT recipients and NKG2A is reportedly involved in GvHD pathogenesis. The percentage of NKG2A^+^ NK cells was significantly reduced in the peripheral blood of GvHD patients after HSCT in comparison with control subsets. Further, these cells were increased in completely recovered GvHD patients compared with partially recovered or active-stage GvHD patients. Therefore, monitoring the frequency of NKG2A^+^ NK cells provides clues for GvHD intervention and treatment. In addition, coculture with NKG2A^+^ NK cells led to decreased IFN-γ secretion as well as proliferation in T cells, indicating that the reduction in NKG2A^+^ NK cells is most likely the cause, rather than the result, of GvHD. The above evidence highlights the importance of NKG2A^+^ NK cells in limiting GvHD by suppressing activated self-reactive T cells (124). In line with this, Kordelas et al. also found a reduction of NKG2A^+^ NK cells in the peripheral blood of GvHD patients after HSCT (125).

## Perspectives

**Figure 1:**
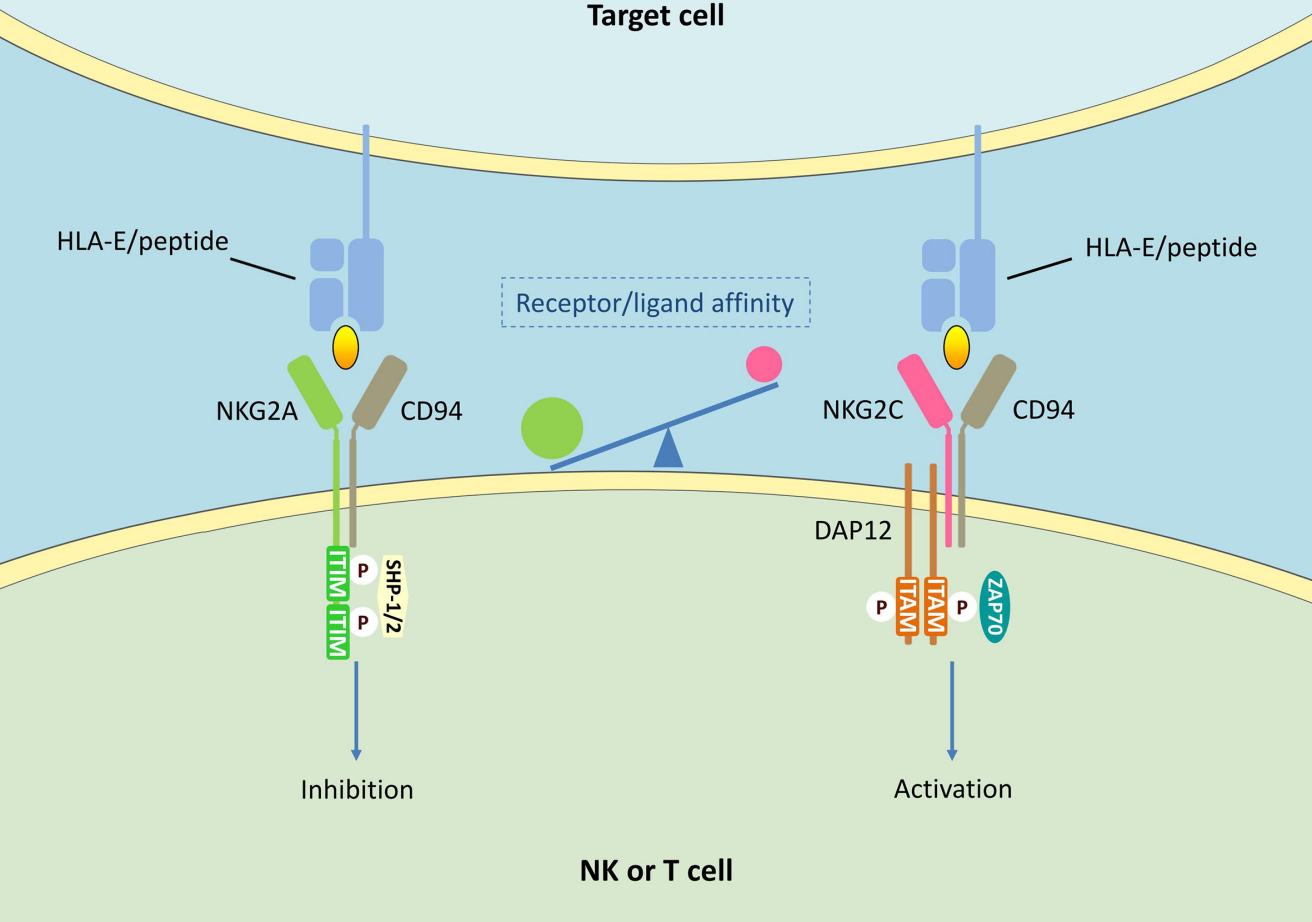
NKG2A/CD94 and NKG2C/CD94 signalings in NK cells and T cells. HLA-E can bind to both NKG2A/CD94 and NKG2C/CD94 receptors. The interaction of NKG2A/CD94 receptor with peptide-presenting HLA­E results in the phosphorylation of ITIMs. Phosphorylated ITIMs are responsible for recruiting and activating intracellular phosphatase SHP-1/2, thus delivering negative signals. CD94/NKG2C heterodimers associates with DAP12 containing immune receptor tyrosine activating motifs (ITAMs), thus activating zeta-chain-associated protein kinase 70 kDa (ZAP70) and delivering activating signals.

A large number of studies have highlighted the critical role of NKG2A in tumors as well as viral infections. It is worth noting that NKG2A is involved in the pathological process of COVID-19 (17–20). The antiviral activity of circulating NK cells and CD8^+^ T cells is markedly decreased during SARS-CoV-2 infection, which leads to severe impairment of the host immune function (126–128). In COVID-19 patients, NKG2A expression is found to be correlated with the severity of disease (17–20). Therefore, anti-NKG2A mAb monalizumab could represent a possible solution for treating COVID-19 patients. Immune checkpoint blockade is one of the most promising ways to activate antitumor immunity. Unlike other known checkpoint molecules such as cytotoxic T lymphocyte-associated antigen-4 (CTLA-4) and PD-1, NKG2A shows selective expression in cytotoxic lymphocytes including NK cells and CD8^+^ T cells. This suggests that the NKG2A-HLA-E axis does not appear to affect the initiation or regulation of anti-tumor immunity, but primarily functions in the final stages of tumor killing. Compared with HLA-E, NKG2A is more suitable as the blockade target of NKG2A-HLA-E axis. Apart from NKG2A, HLA-E also binds to NKG2C. NKG2C is expressed in both NK cells and T cells (129–132), and functions as an activating receptor by associating with the DNAX activation protein of 12 kDa (DAP12) signaling adapter (133) (**Figure 1**). NKG2C and NKG2A recognize mostly overlapping, but partially distinct epitopes on HLA-E (134). Although both NKG2A and NKG2C target HLA-E, the activating receptor shows a much lower affinity for its ligand. Compared with NKG2A, there are some amino acid differences in NKG2C protein, resulting in a 6-fold lower affinity for HLA-E (135, 136). Further, unlike HLA-E, which is expressed on almost all cell surfaces, NKG2A is mainly expressed in tumor lesions. Therefore, blocking NKG2A is more specific. Though NKG2A blockade shows limited effects as a stand-alone therapy, the NKG2A blocking antibody has synergistic effects with other tumor immunotherapies. A central paradigm in current tumor immunotherapy is “combination”, and NKG2A, a modulator of both adaptive and innate immunity, could be an important candidate.

## Author contributions

XW and ZN are responsible for preparation of the published work. HX and ZN are responsible for supervision, review, editing and funding acquisition. All authors contributed to the article and approved the submitted version.

## Funding

The present study was funded by the National Natural Science Foundation of China (No. 82003027), Doctoral Startup Fund of Jining Medical University (No. 2017JYQD24) and Innovation training program for college students of Jining Medical University (No.cx2021065).

## Conflict of interest

The authors declare that the research was conducted in the absence of any commercial or financial relationships that could be construed as a potential conflict of interest.

## Publisher’s Note

All claims expressed in this article are solely those of the authors and do not necessarily represent those of their affiliated organizations, or those of the publisher, the editors and the reviewers. Any product that may be evaluated in this article, or claim that may be made by its manufacturer, is not guaranteed or endorsed by the publisher.
